# ST segment elevation caused by ostial right coronary artery obstruction in infective endocarditis: a case report

**DOI:** 10.1186/s12872-020-01672-1

**Published:** 2020-09-11

**Authors:** Alexander Bolton, Georges Hajj, Laila Payvandi, Christopher Komanapalli

**Affiliations:** 1 Department of Hospitalist Medicine, UnityPoint Health – St. Luke’s Hospital, 1026 A Ave NE, Cedar Rapids, IA 52402 USA; 2 Department of Cardiovascular Medicine, University of Kansas Medical Center, 1400 Cambridge St, BHG600, Kansas City, KS 66160 USA; 3 Department of Cardiology, UnityPoint Health – St. Luke’s Hospital, 1026 A Ave NE, Cedar Rapids, IA 52402 USA; 4 PCI Medical Pavilion, 202 10th Street SE, Suite 225, Cedar Rapids, IA 52403 USA; 5 Department of Cardiothoracic Surgery, The Iowa Clinic at UnityPoint Health – Methodist Medical Center, 1215 Pleasant St, Des Moines, IA 50309 USA

**Keywords:** Acute coronary syndrome, ST elevation myocardial infarction, Endocarditis, Transesophageal echocardiography, Case report

## Abstract

**Background:**

Acute coronary syndrome (ACS) is a rare, but serious complication of infective endocarditis, and diagnosis can be challenging given clinical overlap with other syndromes. A rare cause of ACS in infective endocarditis is mechanical obstruction of the coronary artery. We present the case of a patient with infective endocarditis who developed ST segment myocardial infarction due to occlusion of the right coronary artery ostium by a vegetation.

**Case presentation:**

A 53-year-old female with no prior history of coronary artery disease was transferred to our tertiary care facility for evaluation and treatment of suspected myopericarditis. After transfer she developed inferior ST segment elevations on ECG along with fever and positive blood cultures for methicillin susceptible *Staphylococcus aureus* (MSSA). A transesophageal echocardiogram revealed a vegetation on the aortic valve that intermittently prolapsed into the right coronary ostium. She decompensated from a hemorrhagic brain infarct and subsequently transferred to the intensive care unit. She underwent surgical aortic valve debridement without prior cardiac catheterization given the danger of septic coronary embolization. After a prolonged hospital course with multiple complications, she was able to discharge home, with no neurologic deficits on follow-up.

**Conclusions:**

ACS presents a diagnostic and therapeutic challenge in the setting of infective endocarditis. Careful attention to the history, physical exam and testing can help differentiate infective endocarditis from other conditions sharing similar symptoms. Traditional atherosclerotic ACS management may cause great harm when treating patients with infective endocarditis. The presence of a multidisciplinary endocarditis team is ideal to provide the best clinical outcomes for this population.

## Background

Acute coronary syndrome (ACS) is a rare complication of infective endocarditis, but it confers significant mortality risk when present. A study of 586 cases of endocarditis in Spain revealed that ACS complicated 14 (2.9%) of those cases with 9 deaths. Eight of those deaths were directly attributable to ACS [1]. More recently, a French cohort of 1210 patients with infective endocarditis demonstrated 26 cases (2.2%) of ACS complicating care, with 7 deaths [2]. In these studies, the most common causes of ACS were septic embolization into the affected coronary artery [2], or external compression of the coronary artery by an abscess [1]. This is reflected in multiple case reports published on this topic [3–10].

A seldom documented cause of ACS in infective endocarditis is mechanical obstruction of a coronary artery by a vegetation [2, 11]. We present a case of ST elevation myocardial infarction due to occlusion of the right coronary ostium. We then discuss the challenges in diagnosing her condition, as well as the dilemmas in treatment for ACS in this population.

## Case presentation

**Fig. 1:**
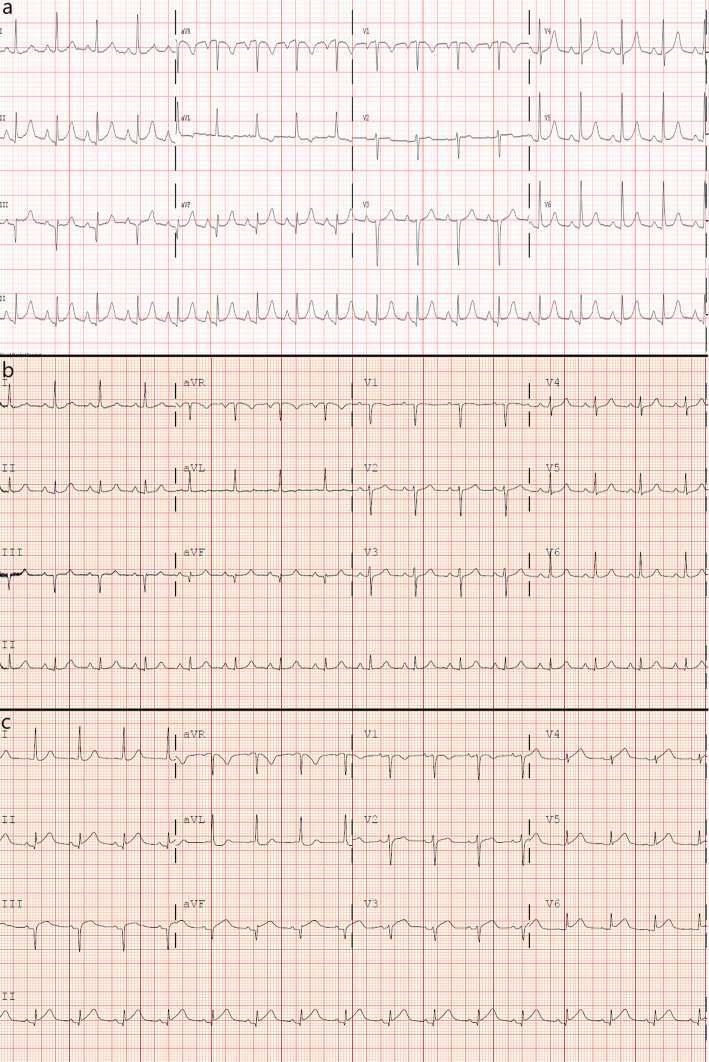
ECGs taken at various stages during the patient’s presentation, demonstrating dynamic changes in ST elevations as well as persistence of Q waves. **a** At initial presentation to the outside hospital. **b** After transfer to our facility. **c** On hospital day 2

A 53 year-old female, with past medical history only notable for hypertension, initially presented to a rural hospital for evaluation of chest pain that was noted to be sharp, radiating towards her back, worse with inspiration and supine positioning, and relieved by sitting forward. This was associated with several days of malaise, poor oral intake, as well as diffuse myalgias. Initial vitals and cardiovascular exam were unremarkable, but ECG on intake demonstrated ST elevations and Q waves in the inferior leads, as well as T wave inversions in leads aVF (Fig. 1a). Troponin I was elevated to 0.49 ng/ml. Initial d-dimer was elevated to 4870 ng/ml, with a CT angiogram of the chest obtained being negative for both pulmonary embolism and aortic dissection. However, coronary calcifications were noted with an empiric diagnosis of ACS. As sublingual nitroglycerin did not provide relief, IV morphine was given along with aspirin 325 mg. IV heparin bolus and infusion were started. The patient was then transferred to our facility for further evaluation and management.

Five hours later, she arrived at our facility. Her physical exam was unchanged. She was noted to have an increased temperature of 38.4 degrees Celsius and elevated troponin I to 2.51 ng/ml. Further lab workup revealed elevations in ESR (42 mm/hr), high sensitivity C reactive protein (296 mg/L), and AST (57 U/L). ECG after transfer demonstrated persistence of the Q waves with resolution of the ST elevations in the inferior leads (Fig. 1b). Transthoracic echocardiogram demonstrated no wall motion nor valvular abnormalities. Given the continued presence and character of her chest pain, she was diagnosed with presumed myopericarditis. With IV heparin discontinued, she was started on naproxen and colchicine, and underwent further workup for a suspected inflammatory process with two separate blood cultures, ferritin (elevated to 2073 ng/ml), procalcitonin (elevated to 2.7 ng/ml), and a full autoimmune panel that was negative.

**Fig. 2:**
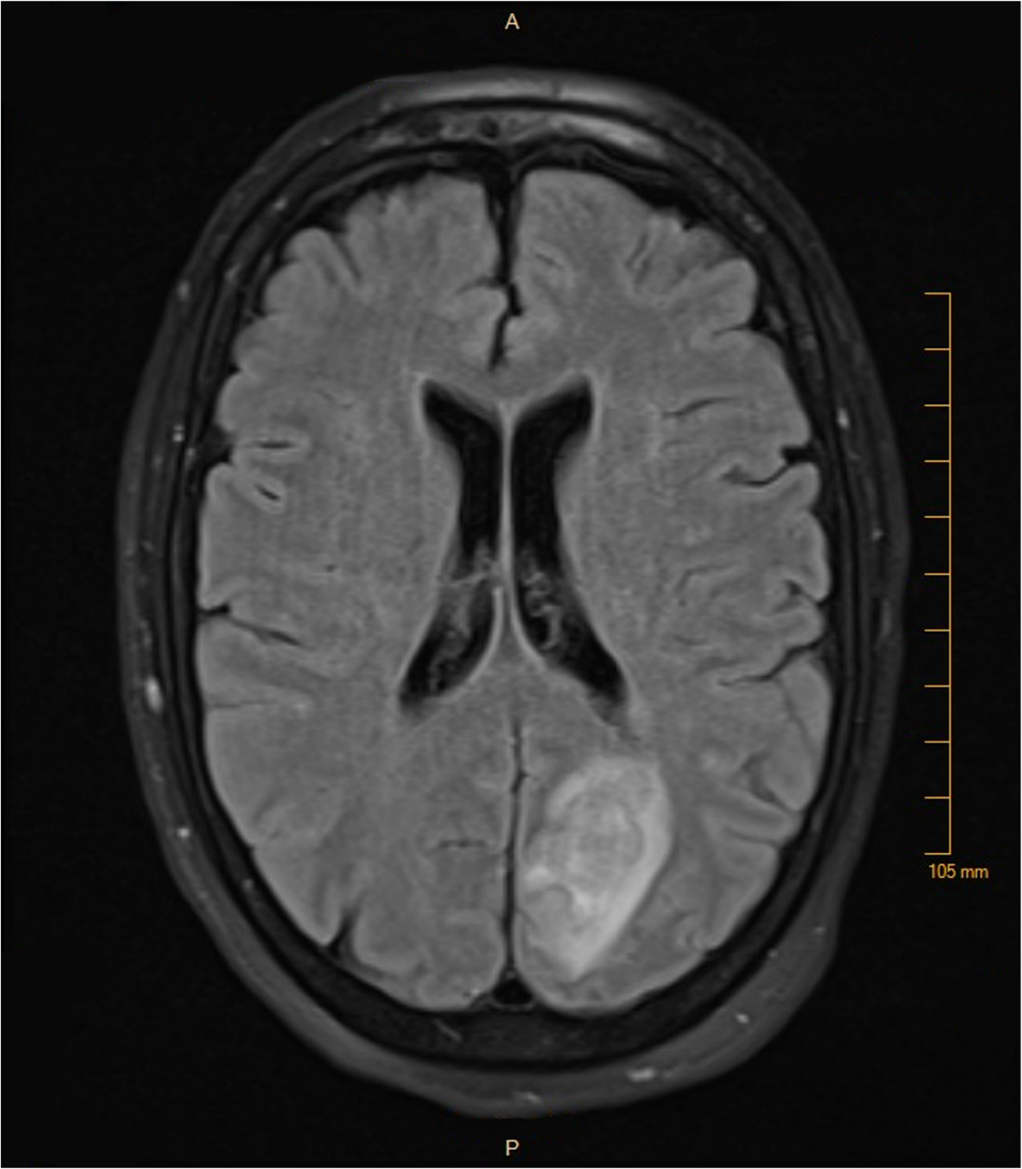
MRI of the patient’s brain on hospital day 3, revealing a left-sided parieto-occipital hemorrhagic infarct

**Fig. 3:**
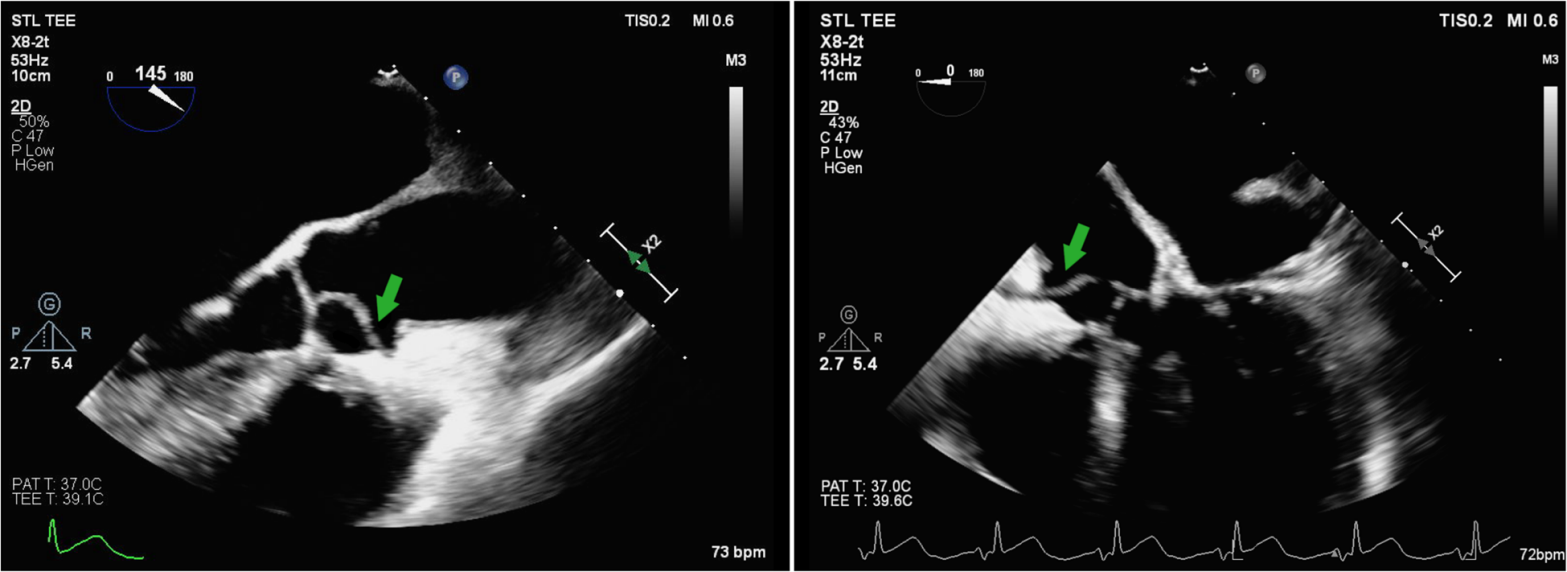
Two transesophageal echocardiography (TEE) views of the aortic valve vegetation (green arrows) showing its prolapse into the right coronary artery ostium


On the evening of hospital day #2, the patient experienced substernal chest pain, altered mental status, and new ST elevations on telemetry monitoring. ECG demonstrated ST elevations highest in the inferior leads, with minor elevation in the anterior as well as lateral leads (Fig. 1c). Upon further questioning, patient was diagnosed with right-sided homonymous hemianopsia. CT and the MRI of the brain revealed left-sided parietal-occipital hemorrhagic infarct (Fig. 2) as well as a small right frontal cortical stroke. Patient was then transferred to the intensive care unit for further monitoring and neurosurgery consultation. Soon after her transfer, blood cultures returned positive for methicillin-susceptible *Staphylococcus aureus* (MSSA). A transesophageal echocardiogram was then obtained, demonstrating a mobile 2.7 cm density attached to the right coronary cusp of the aortic valve that was intermittently prolapsing into the right coronary ostium (Fig. 3, see also Additional files 1-3). No wall motion abnormalities were noted. A diagnosis of infective endocarditis was made. IV nafcillin was started per infectious disease consult recommendations.

**Additional file 1. Transesophageal Echocardiogram Movie 1.** View of aortic valve vegetation with probe at mid-esophageal position, long axis view


**Additional file 2: Transesophageal Echocardiogram Movie 2.** Mid-esophageal long axis view, showing the vegetation entering the right coronary artery ostium.


**Additional file 3: Transesophageal Echocardiogram Movie 3.** Mid-esophageal view with probe at 0 degrees, showing the vegetation entering the right coronary artery ostium.

**Fig. 4:**
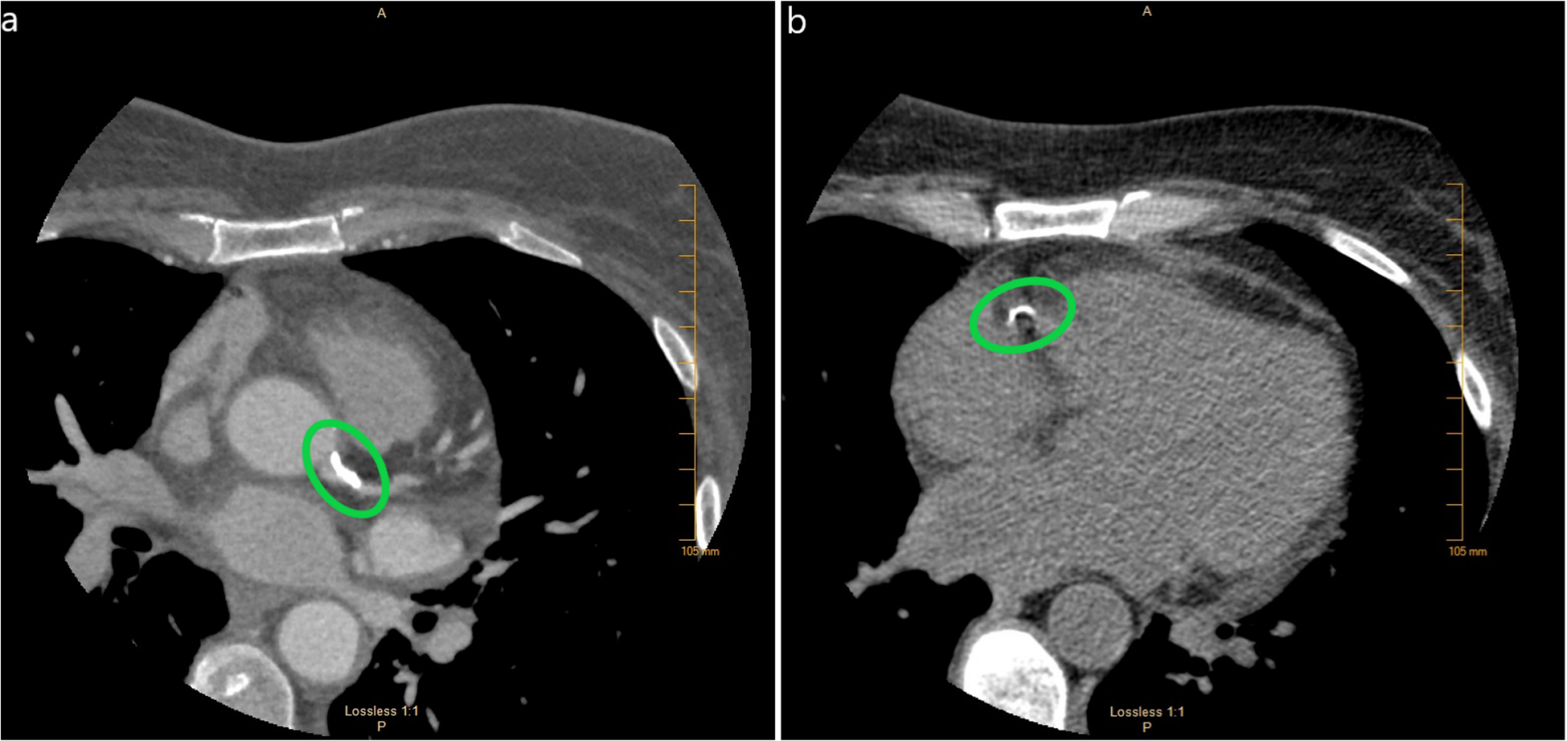
Coronary CT angiogram images showing areas of calcification (green circles). **a** Left circumflex artery **b** Right coronary artery

**Fig. 5:**
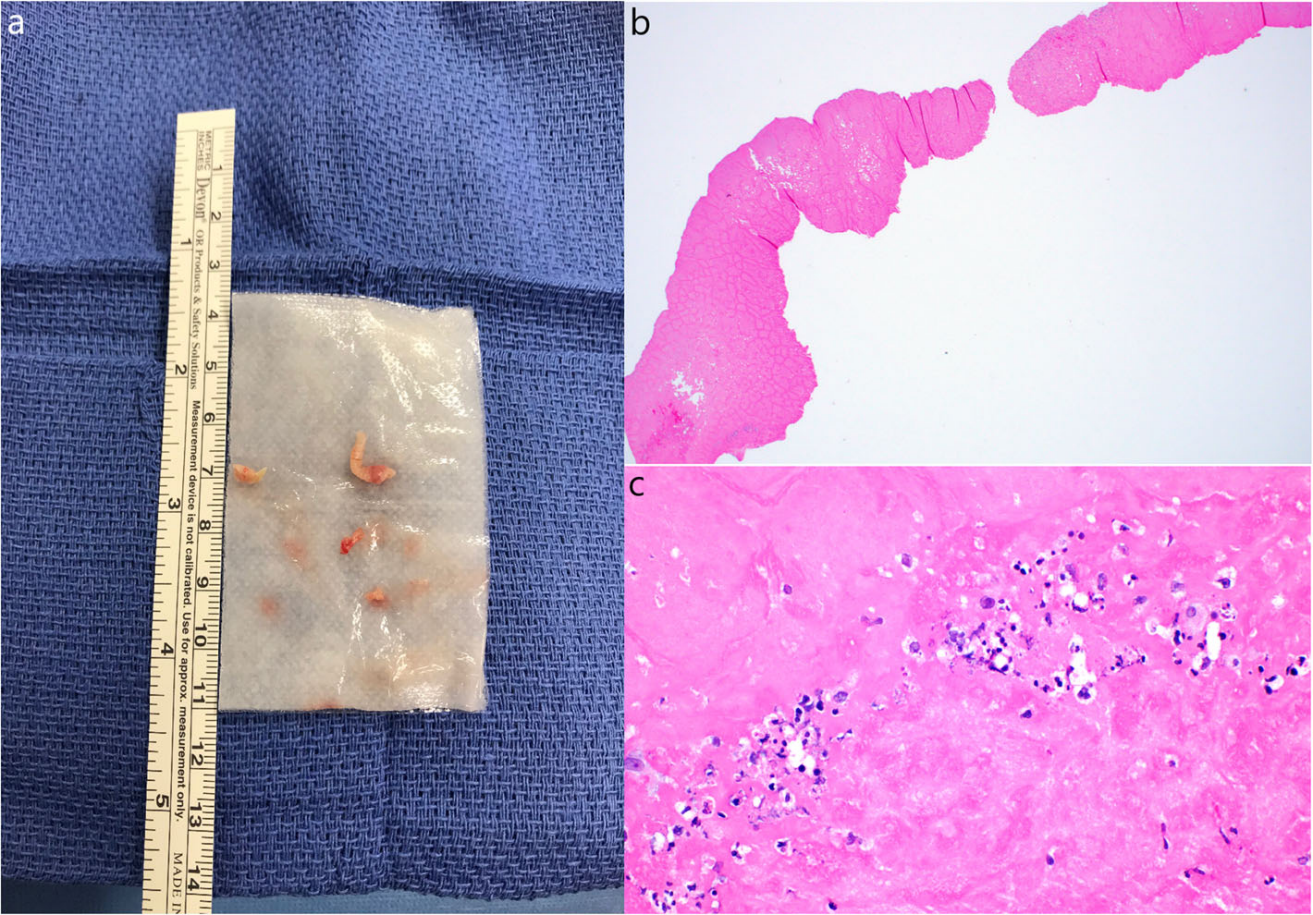
**a** Gross pathology specimen of vegetation. **b** At 2x magnification. **c** At 40x magnification, showing fibrous material and several white blood cells

Cardiothoracic surgery was consulted for assistance in management. As invasive angiography was contraindicated due to her aortic valve endocarditis and hemorrhagic stroke, a coronary CT angiogram was obtained demonstrating significant coronary artery calcifications without obvious focal stenosis. No masses on the aortic valve were noted (Fig. 4a-b). The patient remained hemodynamically stable and free of chest pain for 8 days after transfer to the intensive care unit, with no recurrence of ST elevations on telemetry monitoring. Given her attained stability, as well as the high risk of surgery given her hemorrhagic stroke, the initial plan was for medical treatment with IV antibiotics and no surgical intervention. However, on hospital day #10, patient suffered ventricular fibrillation arrest requiring CPR and defibrillation. After achieving return of spontaneous circulation, the decision was made to proceed with emergent aortic valve surgery given the concern that the origin of cardiac arrest was ischemic in nature from occlusion of the right coronary ostium by the vegetation. A high-risk aortic valve debridement was performed with removal of a 1.0 × 0.4 cm linear vegetation (Fig. 5a-c). Due to the emergent nature of the procedure as well as the lack of obvious focal stenosis on the prior coronary CT angiogram, bypass grafting was not performed.

**Fig. 6:**
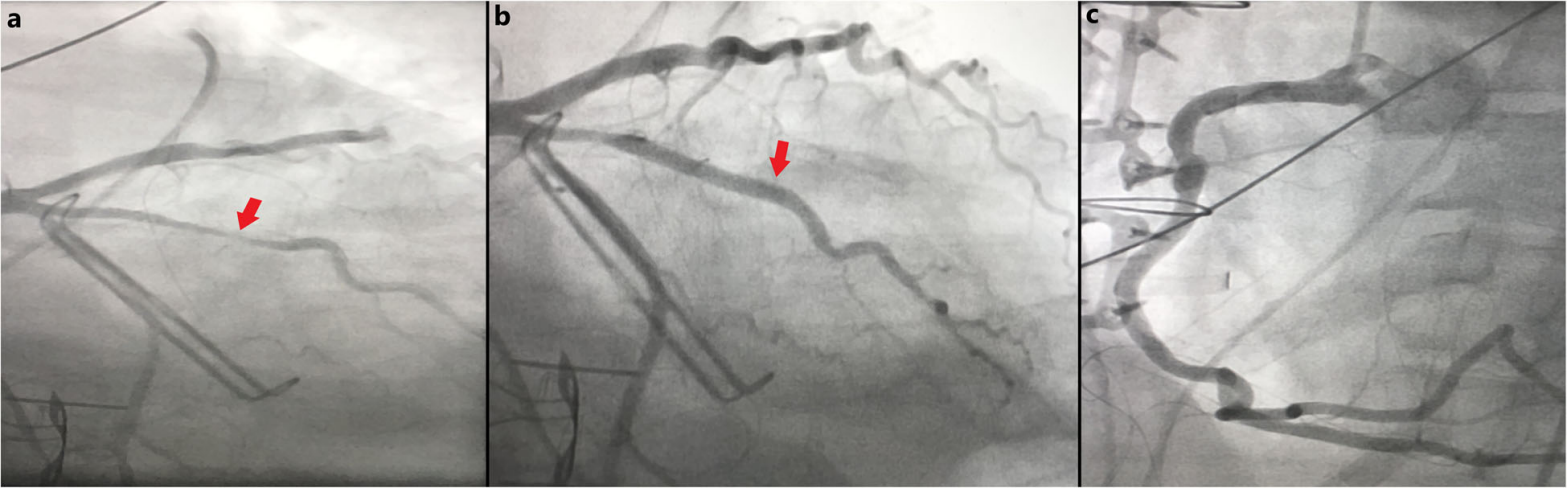
Images from patient’s invasive coronary angiogram on post-operative day #2. **a** Large ramus intermedius artery with 90% focal stenosis (red arrow), **b** The ramus intermedius artery status post deployment of a drug-elating stent, showing restoration of blood flow (red arrow), **c** A patient right coronary artery, with no evidence of arterial disease


Her post-operative course was complicated by ventricular tachycardia on post-operative day 2, requiring urgent coronary angiography and percutaneous coronary intervention with a drug-eluting stent to a 90% stenosis of the ramus intermedius artery. No other significant lesions were seen, including a normal right coronary artery. (Fig. 6a-c, see also Additional files 4-6). After recovery in the intensive care unit, she was transferred to inpatient rehabilitation for further recovery. Her course was further complicated by two readmissions. The first was 2 weeks after surgery for brain abscess managed conservatively with IV antibiotics. The second was 4 weeks after surgery for subsegmental pulmonary emboli managed conservatively without anticoagulation due to her recent hemorrhagic stroke. She developed a drug rash to nafcillin, requiring her antibiotic to be changed to IV vancomycin.

**Additional file 4.** Angiography of the patient’s ramus intermedius artery, showing a 90% stenosis.


**Additional file 5.** Repeat angiography of the ramus intermedius artery after deployment of a drug-elating stent, showing restoration of normal blood flow.


**Additional file 6.** Angiography of the patient’s right coronary artery, showing neither coronary artery disease nor evidence of embolism from the now resected aortic valve vegetation.

**Fig. 7:**
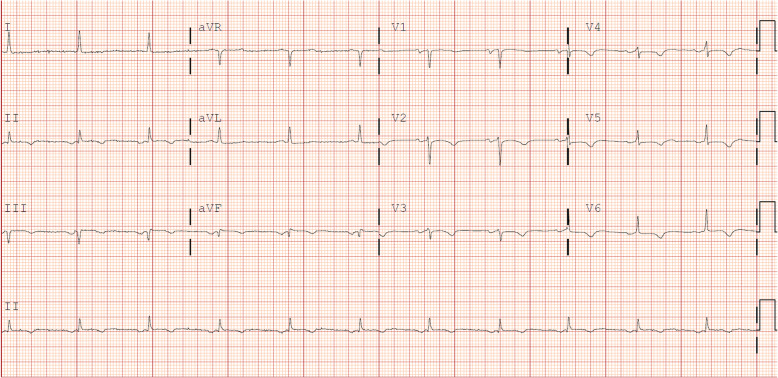
Patient’s ECG taken 2 months after surgery to remove aortic valvular vegetation, showing persistent Q waves in the inferior leads, as well as flat-to-downsloping ST segments and T wave inversions in anterior and lateral leads

Since then, she has fully recovered from her hospitalizations. An ECG on two-month follow-up showed persistent Q waves in the inferior leads, as well as flat-to-downsloping ST segments and T wave inversions in the anterior and lateral leads, suggestive of prior infarction (Fig. 7). However, a transesophageal echocardiogram 1 month after her surgery as well as a transthoracic echocardiogram 3 months after surgery revealed a normally functioning aortic valve with no signs of infection, as well as no wall motion defects. Her most recent encounter was on a one-year follow-up visit to cardiology, where she reported full recovery of her vision and no additional cardiac nor neurologic complications.

## Discussion and conclusions

We present the case of a patient who presented as a transfer to our facility for concerns of myocardial infarction with chest pain and elevated troponin. On our evaluation, the patient also had fever and elevated inflammatory markers, as well as a transthoracic echocardiogram negative for wall motion abnormality. Given the patient’s lack of traditional risk factors for coronary artery disease, the clinical situation was concerning for an atypical cause of her ACS. This concern was further raised once the patient suffered a hemorrhagic stroke. Once positive blood cultures for MSSA were obtained, a transesophageal echocardiogram was performed, identifying the cause of the patient’s ACS as due to infective endocarditis, specifically the mechanical obstruction of the patient’s right coronary ostium by a vegetation. With surgical treatment followed by a full course of IV antibiotics, the patient made a full clinical recovery. Interestingly, while the patient had persistent electrocardiographic changes 2 months after her surgery, echocardiogram failed to demonstrate evidence of RV infarction or other wall motion abnormality at one and 3 months after surgery.

Obstruction of a coronary artery by a vegetation is a rarely documented cause of ACS in the literature. In a study by Tiurin et al. of 104 patients with infective endocarditis in which 11 had ACS, one was noted on autopsy to have a vegetation covering the left main coronary ostium on a native aortic valve [11]. In the study by Roux et al. [2], one case of ACS in the cohort was caused by obstruction of the left main coronary ostium by a vegetation in a patient with a bioprosthetic aortic valve. Since these two cases are reported as part of larger cohorts of infective endocarditis patients, there are no specific clinical nor microbiologic data available to help guide clinicians on patients susceptible to this complication. While septic embolization could be considered as another, more common etiology of our patient's infarction [2], her negative coronary angiogram refutes that possibility.

It is important for clinicians to be aware that for many patients in this cohort, ACS is the first manifestation of infective endocarditis [1, 2, 11], as was the case in our patient. Additionally, many cases of ACS in infective endocarditis occur in the setting of aortic valve vegetations [1–5, 7]. Given the myriad ways infective endocarditis presents, it can easily be confused for myocarditis or pericarditis, given both present with fever, elevated inflammatory markers, and ST elevations on ECG. However, on retrospective review of our case, there were findings suggestive of the possibility of infective endocarditis. Compared to pericardial disease presenting with diffuse ST elevations and PR depressions, ST elevations were most prominent in the inferior leads for our patient, indicating the territory affected by the blockade of the right coronary ostium. Once blood cultures were positive, subsequent physical examination of the patient found poor dentition and faint macules on her palms consistent with Janeway lesions. This underscores the importance of careful physical exam and testing to differentiate between disease processes that appear similar.

Most patients in observational cohort studies with ACS and infective endocarditis do not have pre-existing coronary artery disease [1, 2]. However, the presentation of ACS in this population can influence clinicians to swiftly apply traditional management strategies for atherosclerotic disease. The problem is that typical ACS management may potentiate adverse outcomes in the subset of infective endocarditis cases. Due to risk of septic embolism and subsequent hemorrhagic complications, thrombolysis is generally contraindicated in infective endocarditis, as multiple case reports have shown an increase in intracranial hemorrhage that often leads to morbidity and mortality [5, 6, 8, 12]. Percutaneous interventions in areas of luminal narrowing, either with balloon angioplasty or with stent implantation, carry their own risks of infection or mycotic aneurysm formation at the site of intervention. This is likely due to small vegetative emboli being fractured and crushed against the blood vessel wall during the procedure [7, 9].

Given the existing limitations of evidence for management of ACS in infective endocarditis, as well as the diversity of causes for ACS in this specific population, an endocarditis team comprising the specialties of interventional and non-interventional cardiology, cardiothoracic surgery and infectious disease is necessary to thoroughly assess the patient and provide recommendations for the best individualized therapeutic approach. In cases where percutaneous intervention is the best option, there has been success using catheter aspiration thrombectomy to restore flow, followed by stenting only if required to maintain patency. This approach minimizes risk of both aneurysm formation and bleeding complications from antiplatelet therapy [7, 10]. As in our patient, a surgical approach may be considered given a mechanical obstruction from the vegetation as cause of their ACS. Depending on the severity of the patient’s condition, the decision to operate may be difficult. Surgery offers the advantage of having both a definitive management of infective endocarditis as well as options for revascularization, if needed.

In conclusion, acute coronary syndrome is a rare but serious complication of infective endocarditis, and the combination of prompt diagnosis with timely but appropriate management is required for the best outcome. As ACS can be the first manifestation of infective endocarditis, IE should be considered early in presentations of ACS when accompanied by fever and elevated inflammatory markers, especially if the patient lacks traditional risk factors for coronary artery disease. There can be strong clinical overlap between infective endocarditis and other conditions. Therefore, careful physical examination along with thorough laboratory assessment is critical to ascertain the correct diagnosis. ACS in infective endocarditis has several possible etiologies. To determine the optimal treatment, an interdisciplinary endocarditis team is ideal to choose the optimal management strategy on a case-by-case basis. More experience and research is necessary to generate evidence-based recommendations for diagnosis and treatment of ACS in infective endocarditis.

## Patient perspective

“First of all, before this happened I was never sick. A slight cold was it. Maybe a little indigestion but never anything serious. So when my back started to hurt and I had an upset stomach I decided it was from my day’s activities. Sunday morning when my alarm went off for work I could hardly get out of bed. I called a co-worker to cover for me and went back to bed. I hadn’t called in to work the entire 10 years I had been there. Monday morning I forced myself to go to work. I was having lots of pain in my back and chest. I finally found someone to come in and my sister took me to the hospital. I thought I would get some pain meds and go home. That didn’t happen. I wouldn’t see home for almost a month.

There are many things that happened that I don’t remember but I have read about from the posts my family had written on [Facebook]. I do remember things going well and moving out of [the] ICU only to have my alarms go off and my heart beating out of my chest and back to the ICU I go. I remember after the first time it happened the panic I would feel each time my heartbeat was not regular. I remember the support and sympathy from the nurses every time they couldn’t get blood and had to try 3–4 times. I remember telling my family and friends that it would be okay, we just had to think positive. I remember not being able to remember the simple things. I think for me that was harder than the pain.

Through everything my family support was amazing. Now I’m physically back to normal. I have no lasting effects from my journey – physically anyway. My mental state will forever be changed. No one can be that close to death and not be changed. My faith has been restored and I’ve been given a second chance at life. Hopefully my experience will give someone else another chance.”

## Data Availability

Not applicable.

## Abbreviations

ACS: Acute coronary syndrome
AST: Aspartate transaminase
CPR: Cardiopulmonary resuscitation
CT: Computed tomography
ECG: Electrocardiogram
ESR: Erythrocyte sedimentation rate
IV: Intravenous
MRI: Magnetic resonance imaging
